# Clinical Characteristics and Outcomes of Dilated Cardiomyopathy in Chinese Children: A Single-Center Retrospective Study

**DOI:** 10.3390/children11080992

**Published:** 2024-08-15

**Authors:** Cheng Chen, Yanyun Huang, Danyan Su, Suyuan Qin, Bingbing Ye, Yuqin Huang, Dongli Liu, Yusheng Pang

**Affiliations:** 1 Department of Pediatrics, The First Affiliated Hospital of Guangxi Medical University, Nanning 530021, China; yfy004118@sr.gxmu.edu.cn (C.C.); yfy107391@sr.gxmu.edu.cn (Y.H.); sudanyeal@sr.gxmu.edu.cn (D.S.); yfy003299@sr.gxmu.edu.cn (S.Q.); yfy004293@sr.gxmu.edu.cn (B.Y.); yfy107393@sr.gxmu.edu.cn (Y.H.); dongli.liu@sr.gxmu.edu.cn (D.L.); 2 Difficult and Critical Iillness Center, Pediatric Clinical Medical Research Center of Guangxi, Nanning 530021, China

**Keywords:** dilated cardiomyopathy, children, outcome, risk factors

## Abstract

Background: The reported outcomes of pediatric dilated cardiomyopathy (DCM) have varied across studies. There are few outcome data concerning DCM in Chinese children. Therefore, we conducted a retrospective study to describe clinical features and determine risk factors for poor outcomes in children with DCM. Methods: We enrolled 121 children with DCM in our hospital from 2003 to 2021. General information and laboratory and echocardiographic data were collected and analyzed. Cox regression analysis was performed to determine risk factors for poor outcomes. Results: This study included 121 patients (69 males and 52 females). The median age at diagnosis was 10.8 years, and the follow-up time was 10.0 months. Eighty-two patients (67.8%) exhibited cardiac function classes III–IV at the time of diagnosis. Tachypnea was the most common symptom (78.5%). In echocardiography, the mean left ventricular end-diastolic dimension z score was 7.36 ± 2.73, and the left ventricular ejection fraction z score was −6.58 ± 2.17. The 1-, 2-, and 5-year survival rates were 51.2%, 43.8%, and 32.2%, respectively. Cox analysis revealed that cardiac function classes III–IV (hazard ratio [HR] = 1.801, 95% confidence interval [95% CI] = 1.030–3.149, *p* = 0.039) and calcium levels (HR = 0.219, 95% CI = 0.084–0.576, *p* = 0.002) were predictors of poor outcomes in children with DCM. Conclusions: Children with DCM are at high risk of death. Cardiac function class III–IV and calcium levels were related to the prognosis of pediatric DCM patients.

## 1. Introduction

Dilated cardiomyopathy (DCM) is a life-threatening disorder characterized by left ventricle dilation and impaired contractility [1]. It is the most common form of pediatric cardiomyopathy [2]. Epidemiologically, in the United States, DCM incidence was 0.57/100,000 per year for children aged 0–18 years [3]. Approximately 64% of patients with DCM died or underwent a cardiac transplant within 1 year of their first admission [4]. Despite substantial advances in treatment, the prognosis for DCM patients, especially children, remains poor [5]. The reported outcomes of DCM in children vary widely depending on the medical condition of patients in the region. One single-center retrospective study from India reported a 5-year survival rate of 59% [6], while another study from the United States estimated a 5-year survival rate of 83% with heart transplantation [7]. According to a population-based study from Australia, the survival rates of children with DCM who died or underwent cardiac transplantation over 1 year and 5 years were 72% and 63%, respectively [8]. However, few data have addressed DCM outcomes in Chinese children. Previous studies have provided great insights into the risk factors for poor outcomes in children with DCM. Age at diagnosis, male sex, familial DCM, heart failure, heart rate, left ventricular fractional shortening (LVFS) z score, left ventricular ejection fraction (LVEF) z score, uric acid, serum cholesterol, atrial filling pressure, and mixed venous saturation are considered predictors of poor outcomes in pediatric patients with DCM [3,4,8,9]. However, the results vary between different studies. A better understanding of the clinical features and risk factors for poor outcomes in children with DCM may help improve individual risk stratification and develop better treatment strategies. Therefore, we conducted this retrospective single-center study in a region tertiary hospital in Southwestern China to describe clinical features and identify risk factors for poor outcomes in children with DCM.

## 2. Materials and Methods

### 2.1. Patients and Definitions

This study was performed at the First Affiliated Hospital of Guangxi Medical University. Patients with DCM admitted from March 2003 to September 2021 were consecutively enrolled. DCM was defined as a dilation and systolic dysfunction of the LV. LV dilation was defined as an LV end-diastolic dimension (LVEDD) exceeding two standard deviations (SDs) of the body surface area-adjusted mean value (i.e., z score > 2). LV systolic dysfunction was defined as an LVEF or LVFS lower than 2 SDs for a reference population of the same age (i.e., z score < 2) [1,10]. The inclusion criteria for patients were as follows: (1) met the diagnostic criteria for DCM; (2) aged < 18 years; (3) had complete and traceable clinical data. Patients with DCM caused by arrhythmia, hypertensive heart disease, congenital heart disease, cardiac valve disease, ischemic heart disease, infection, inherited metabolic disease, and drugs were excluded. The cardiac function class was confirmed at the time of presentation by specialist pediatric cardiologists using the New York Heart Association (NYHA) class (age > 1 year) or modified Ross score (age ≤ 1 year) [11,12]. Cardiac function I was defined as NYHA class I or modified Ross scores of 0–2 points, cardiac function II as NYHA class II or modified Ross scores of 3–6 points, cardiac function III as NYHA class III or modified Ross scores of 7–9 points, and cardiac function IV as NYHA class IV or modified Ross scores of 10–12 points. The study protocols were in accordance with the Helsinki Declaration. Written informed consent from parents was obtained. Ethical approval was obtained from the Ethics Committee of the First Affiliated Hospital of Guangxi Medical University (NO. 2022(KY-E-007)).

### 2.2. Data Collection

General data, including age, sex, race, family history of DCM, body mass index, cardiac function class, heart rate, systolic blood pressure (SBP) and diastolic blood pressure (DBP), main syndromes and signs, symptom duration, duration of first hospitalization, admission to the intensive care unit, and multiple hospitalizations (≥2 visits), were obtained from the medical records. We collected laboratory data, including white blood cells, red cell distribution width, hemoglobin, troponin I, creatine kinase-MB, electrolytes, serum creatinine, blood urea nitrogen, and uric acid levels, within 24 h after the first admission. Each patient underwent echocardiography at admission. We recorded echocardiographic parameters such as LVEDD, left ventricular end-diastolic posterior wall thickness, left ventricular end-systolic dimension (LVESD), LVEF, and LVFS. These echocardiographic measurements were normalized by the z score to adjust for body surface area and age. The treatments administered to patients, including digoxin, β-blockers, angiotensin-converting enzyme inhibitors (ACEIs), and diuretics, were also noted. Follow-up was performed via medical records or telephone contact. The endpoint was all-cause death during follow-up.

### 2.3. Statistical Analysis

The Shapiro–Wilk test was used for the normality test. Categorical variables were expressed as frequencies (percentages) and intergroup comparisons were performed with chi-square tests or Fisher’s exact test. Quantitative variables are presented as the mean ± SD (normal distribution) or median with an interquartile range (skewed distribution). Student’s *t*-test or the Mann–Whitney U test was used to compare quantitative data between the groups, as appropriate. A Kaplan–Meier curve was plotted for survival analysis and analyzed by the log-rank test. We performed Cox regression analysis to determine risk factors for poor outcomes in children with DCM and calculate hazard ratios (HRs) and 95% confidence intervals (95% CIs). Factors with a *p*-value < 0.1 in the univariate Cox regression were included in the multivariate Cox regression model. The cutoff value for calcium concentrations was determined using the receiver operating characteristic (ROC) curve. *p* < 0.05 was considered to indicate statistical significance. The data were analyzed with SPSS software (version 24.0 for Windows, SPSS, Inc., Chicago, IL, USA).

## 3. Results

### 3.1. Baseline Characteristics

**Figure 1:**
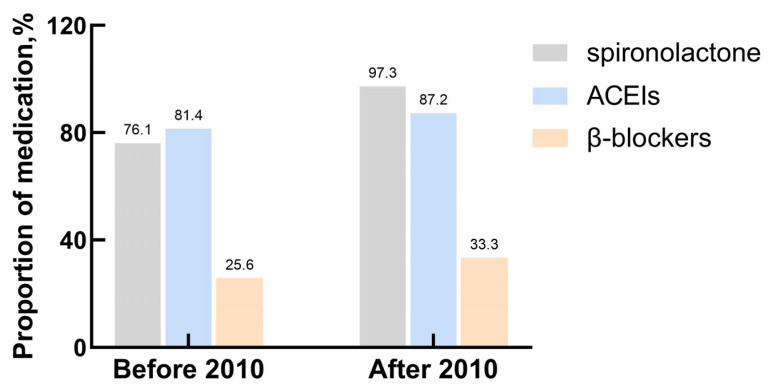
The explanation of prescription rates of major drugs.

This study involved 121 patients, including 69 males and 52 females. The median age at diagnosis was 10.8 (5.3–14.0) years. Thirty-nine patients (32.2%) exhibited cardiac function classes I–II, and eighty-two (67.8%) had cardiac function classes III–IV at the time of diagnosis. Ten (8.3%) patients had a family history of DCM. Tachypnea was the most common symptom detected in 78.5% of patients, followed by fatigue, hepatomegaly, edema, and chest distress. The median symptom duration was 1 (0.5–3.0) month, and the median length of the first hospitalization was 9 (6.0–15.0) days. Fifty-three (43.8%) patients were admitted to the ICU when diagnosed. Fifty-one (42.1%) patients needed multiple hospitalizations due to poor disease control. At the time of diagnosis, 94.2% of the patients (114/121) were prescribed a loop diuretic and digoxin, and 91.7% (111/121) were prescribed spironolactone, with 85.1% (103/121) receiving ACEIs, 50.4% (61/121) using intravenous inotropes, and 30.5% (37/121) receiving β-blockers. The prescription rates of spironolactone increased significantly after 2010 compared to before 2010, while the prescription rates of ACEIs and β-blockers remained stable (Figure 1). Compared with survivors, patients who died from all causes had a higher heart rate and lower SBP and DBP (both *p* < 0.05). The median follow-up period was 10 (3.0–36.5) months. A summary of the baseline characteristics is shown in Table 1.

### 3.2. Laboratory and Echocardiographic Examination

The results of the laboratory and echocardiographic examinations at the time of diagnosis are shown in Table 2. Echocardiography revealed an enlarged LV dimension, with a mean LVEDD z score of 7.36 ± 2.73 and a mean LVESD z score of 10.42 ± 3.35. In addition, patients in the death group had a greater LVESD z score than the survivors (*p* = 0.038). The mean LVEF z score was −6.58 ± 2.17, and the median LVFS z score was 9.45 (−11.55 to −6.36), indicating severely reduced systolic function. A lower serum calcium concentration was observed in the death group than in the survivor group (*p* = 0.002). However, neither group significantly differed in white blood cell count, red cell distribution width, hemoglobin, troponin I, creatine kinase-MB, sodium, potassium, uric acid, blood urea nitrogen, or serum creatinine levels (*p* > 0.05).

### 3.3. Survival Analysis

**Figure 2:**
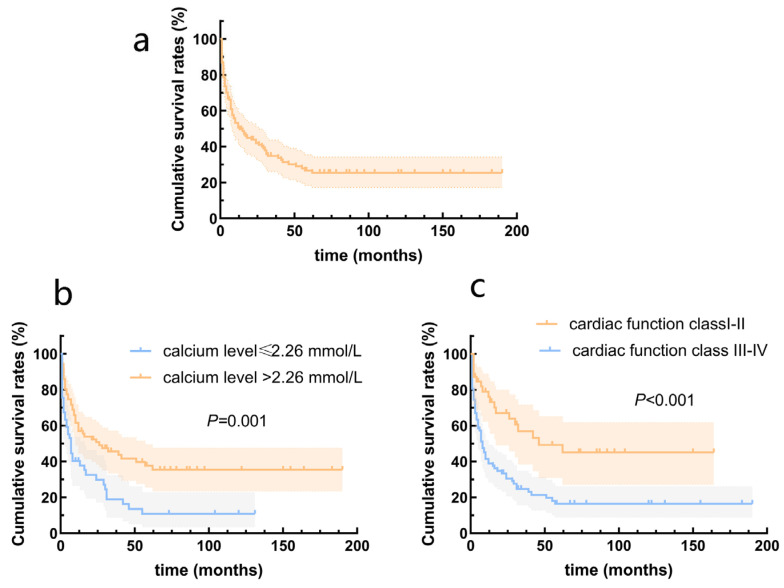
Survival rates from the Kaplan–Meier estimates in patients with dilated cardiomyopathy. (**a**) Total patients; (**b**) patients grouped by serum calcium levels; (**c**) patients grouped by cardiac function class.

**Figure 3:**
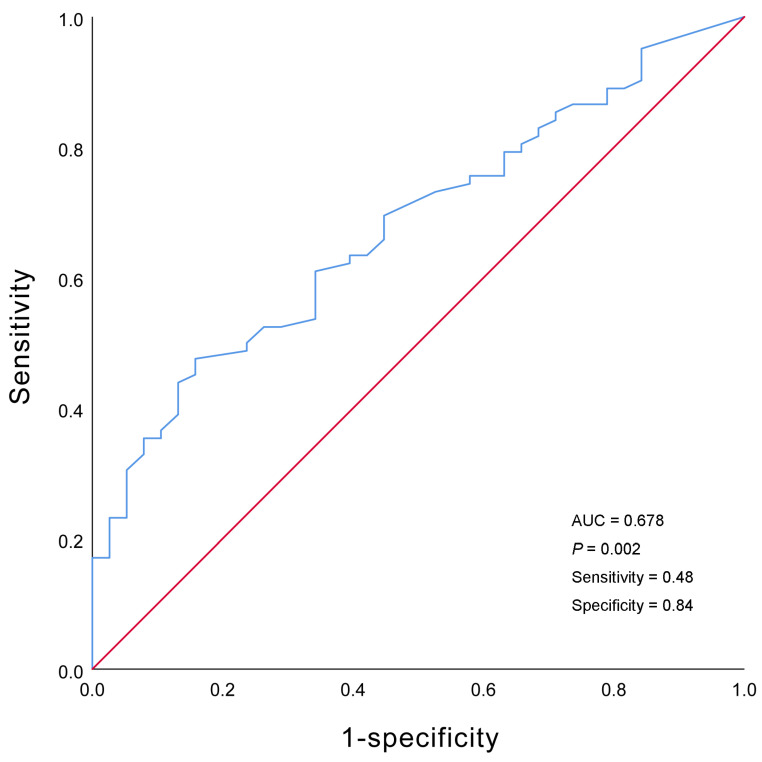
Receiver operating characteristic curve of the serum calcium concentration.

**Figure 4:**
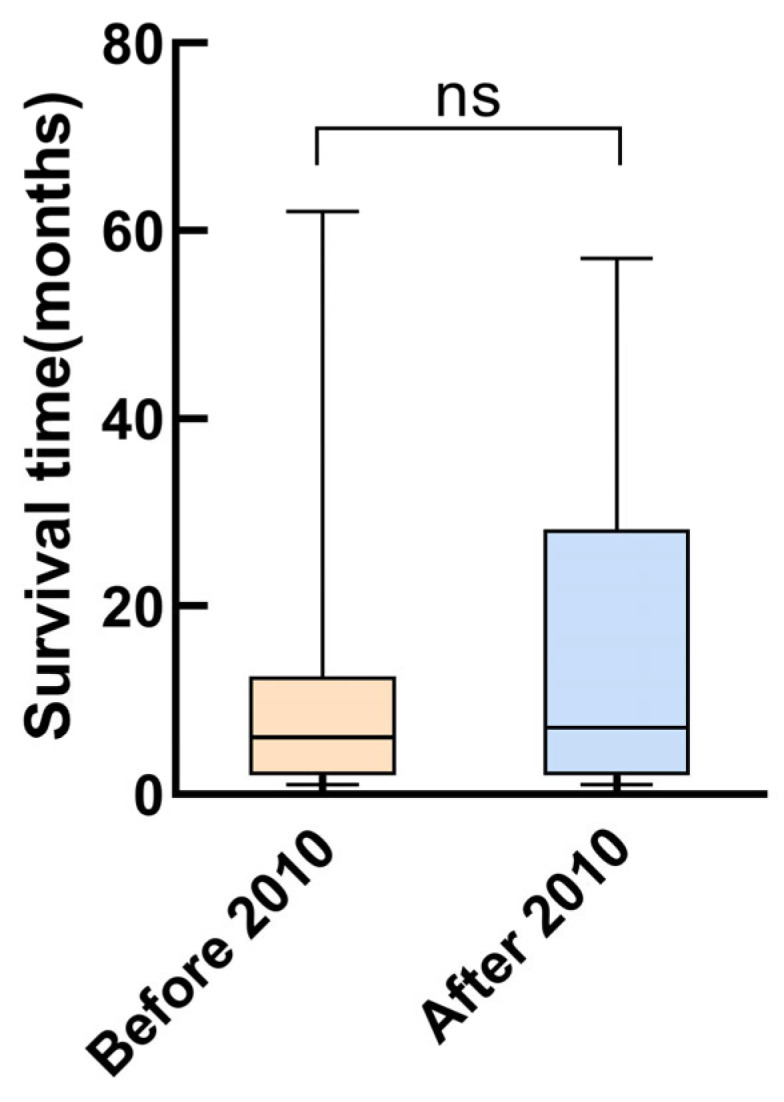
Survival time of the patients who died from all causes at different recruitment periods.

The survival rates at one, two, and five years were 51.2%, 43.8%, and 32.2%, respectively. The highest mortality was observed during the first year after diagnosis, at which time 48.8% of the children reached the endpoint (Figure 2a). Univariate Cox analysis revealed that SBP (HR = 0.968, 95% CI = 0.949–0.987, *p* = 0.001), DBP (HR = 0.977, 95% CI = 0.959–0.966, *p* = 0.018), calcium level (HR = 0.222, 95% CI = 0.084–0.574, *p* = 0.002), and cardiac function classes III–IV (HR = 2.516, 95% CI = 1.488–4.255, *p* = 0.001) were predictors of death in patients with DCM. According to the multivariate Cox analysis, only cardiac function classes III–IV and calcium levels remained significant (Table 3). Patients with cardiac function classes III–IV at diagnosis were 1.801 times more likely to die than those with cardiac function classes I–II (HR = 1.801, 95% CI = 1.030–3.149; *p* = 0.039). Increased calcium concentration of 1 mmol/L reduced the risk of death by 78.1% (HR = 0.219, 95% CI = 0.084–0.576, *p* = 0.002). The area under the curve for calcium concentration was 0.678 to distinguish between patients who died from all causes and survivors (95% CI = 0.581–0.775, *p* = 0.002; Figure 3). The sensitivity was 0.48, and the specificity was 0.84. The cutoff value was 2.26 mmol/L. Patients were categorized into a calcium concentration cutoff group (≤2.26 mmol/L) and a calcium concentration >2.26 mmol/L group. According to the Kaplan–Meier survival analysis, patients with a calcium concentration ≤2.26 mmol/L had a lower cumulative survival rate (*p* = 0.001; Figure 2b). Patients with cardiac function classes III–IV had a significantly lower survival rate than those with cardiac function classes I–II (*p* < 0.001; Figure 2c). Among the 83 patients who died from all causes, the median survival time (defined as the time from diagnosis to death) for those diagnosed before 2010 was 6.0 (2.0–12.5) months, while for those diagnosed after 2010 it was 7.0 (2.0–28.3) months (Figure 4).

## 4. Discussion

We retrospectively analyzed the clinical features and outcomes of DCM patients in a large academic tertiary center in Guangxi, China. The results revealed that most patients were symptomatic at the time of diagnosis and usually severely ill. For instance, 67.8% of the patients presented with cardiac function classes III–IV and the majority needed medication. The mortality rate was relatively high in children with DCM admitted to the hospital. Cardiac function classes III–IV and serum calcium levels were found to be independent predictive factors of death in pediatric DCM patients.

The reported survival rates of DCM patients have varied across studies. One-year survival rates of 63–90% and 5-year survival rates of 34–83% have been described in institutional reviews [3,4,5,7,9,13,14]. In our cohort study, the 1-year survival rate was 51.2%, and the 5-year survival rate was 32.2%, suggesting a greater risk of death than in other studies. One possible explanation for this difference might be that none of the patients in our study underwent cardiac transplantation. Cardiac transplantation is a final therapeutic strategy for DCM patients and can improve patient prognosis. One study reported that 1- and 5-year survival rates were 90% and 83%, respectively, with heart transplants [7]. However, the percentage of patients free from “cardiac death” (death or cardiac transplant) was only 70% at 1 year and 58% at 5 years. Another study reported by Hollander et al. [4] showed that the total survival rate at 1 year was 84.5% in 83 pediatric patients with DCM. However, the transplantation-free survival rate was only 46% at 1 year, which was similar to our total survival rate in the population without cardiac transplants. Another reason for the high mortality could be that most of the patients in this study were severely ill. Our hospital is a tertiary-care medical institution that receives patients with severe conditions from local hospitals. In this study, the majority (67.8%) of patients presented with advanced heart failure at the time of onset. Almost half of our patients (43.8%) had to be admitted to the intensive care unit, indicating a poor prognosis.

The NYHA class is usually used to evaluate the severity of HF. However, this grading system is based on the limitations of physical activity in adults and may not apply to children, especially infants. Therefore, we used a modified Ross score rather than the NYHA class in patients aged ≤1 year. We concluded that cardiac function III–IV at the time of diagnosis increased the risk of all-cause death by 80.1%, which was consistent with previous studies. Castelli et al. [15] reported that a high NYHA class was associated with cardiovascular mortality and heart transplantation. Similarly, Christ et al. [16] reported that a high NYHA class increased the risk of death or heart transplant in patients with idiopathic DCM. Research has also shown that primary outcomes at 1 year are predicted by the NYHA class in both familial and sporadic DCM patients [17]. Heart failure at the time of diagnosis was correlated with a 3.18-fold increased risk of transplantation or death in patients with both familial DCM and idiopathic DCM [18]. These results revealed that patients with a higher cardiac function class are more severely ill and likely to have poor outcomes. A key goal of DCM therapy may be to reduce heart failure.

To the best of our knowledge, this is the first study to show the prognostic value of serum calcium concentration in DCM patients. Calcium is a crucial mediator of cardiac contraction and diastole, playing a key role in regulating excitation–contraction coupling [19]. In recent years, several studies have reported that hypocalcemia is related to poor prognosis in patients with HF. Liu et al. [20] demonstrated that hypocalcemia predicted cardiac readmission and death within 1 year. Additionally, Jensen et al. [21] proposed that an imbalance in calcium homeostasis results in a greater mortality rate in chronic heart failure patients. Furthermore, Miura et al. [22] reported that hypocalcemia was an independent risk factor for death in patients with heart failure and chronic kidney disease. However, the etiologies of heart failure are quite diverse. The primary causes of heart failure were not considered in previous studies. To date, serum calcium concentration has not been reported to predict the prognosis of DCM patients. In this study, we found a negative correlation between high serum calcium levels and poor prognosis in patients with DCM. Specifically, for each unit increase in calcium concentration, the risk of death falls by 78.1%. Patients with calcium levels ≤2.26 mmol/L had worse survival outcomes than those with calcium levels >2.26 mmol/L. Extracellular calcium concentration may affect myocardial contractility by influencing the membrane potential of cardiac cells [23]. Serum calcium influxes into the cytosol when the membrane potential changes, leading to an increased release of calcium from the sarcoplasmic reticulum. In such circumstances, calcium binds to cardiac troponin C and causes the myocardium to contract [24,25]. Therefore, low serum calcium levels may reduce cardiac contractility. Yang et al. [26] reported that cardiac systolic function rapidly improved in a 56-year-old DCM patient with recurrent hypocalcemia after serum calcium level restoration, indicating that hypocalcemia is an important cause of DCM. However, the mechanism responsible for calcium’s impact on DCM has not been elucidated.

Some predictors of poor prognosis for patients with DCM in the literature are age at diagnosis, male sex, the LVFS z score, the LVEF z score, heart rate, and BP at diagnosis [3,8,27,28,29,30]. However, age at diagnosis, sex, the LVFS z score, and the LVEF z score were not related to the risk of death in our study. Consistent with findings in the literature [30,31], univariate Cox regression analysis revealed that SBP and DBP were protective factors against death in patients with DCM. This outcome is likely because patients with low BP are more likely to have lower cardiac output and neurohormonal activation, which may exacerbate their symptoms [32,33]. However, the correlation was not significant according to the multivariate Cox regression model, likely due to the effects of confounding factors.

DCM treatment is aimed at alleviating symptoms, improving quality of life, preventing disease progression, and increasing survival. Most patients in our cohort received medical therapy, which has been shown to decrease mortality and cardiac remodeling. This therapy included ACEIs, β-blockers, and mineralocorticoid receptor antagonists [34,35]. Digoxin, a medicine commonly used for heart failure and DCM, can reduce hospitalization and mortality in patients at high risk of heart failure [36,37]. In the present study, 94.2% of the patients received loop diuretics and digoxin, 91.7% received spironolactone, 85.1% received ACEIs, and 30.5% received β-blockers. These results were comparable with other studies [8,14,29]. In our study, the prescription rates of spironolactone increased significantly after 2010, while the prescription rates of ACEIs and β-blockers remained stable. The survival time in this study showed no evident temporal trend toward improvement. No significant associations were observed between the use of medical drugs and patients’ prognosis. Although medical therapy has been reported to be beneficial for children with DCM in the past, we could not conclude from the current study that medical therapy modified the outcome of children with DCM. The children included in this study were quite ill and at high risk of death, and may have benefited from effective early treatment.

### Limitations

This study has several limitations. First, selection bias was inevitable due to the retrospective nature of the study. Second, this was a single-center study with a relatively small sample size. Third, genetic tests were not routinely available in our study. Due to the limited availability of gene data, gene analysis was not performed in this study. Finally, DCM etiologies were heterogeneous in our study. However, too few patients were included in the subgroup analysis.

## 5. Conclusions

In summary, the findings of this study demonstrated that children with DCM are at high risk of death, with the highest risk occurring during the first year after diagnosis. Survival was lower in patients with cardiac function III–IV and low serum calcium levels at the time of diagnosis. Efficient risk stratification, early individualized therapy, and close outpatient observation may improve these patients’ outcomes.

## Figures and Tables

**Table 1:** Baseline characteristics of patients with dilated cardiomyopathy.

Parameters	All Patients (*n* = 121)	Death (*n* = 83)	Survivors (*n* = 38)	*p*-Value
Age at diagnosis (years)	10.8 (5.3–14.0)	10.60 (5.1–13.7)	10.95 (5.5–15.2)	0.569
Male, *n* (%)	69 (57.0)	43 (51.8)	26 (68.4)	0.087
Race, *n* (%)				0.333
Han	58 (47.9)	41 (49.4)	17 (44.7)	
Zhuang	57 (47.1)	38 (45.8)	19 (50.0)	
Others	6 (5.0)	4 (4.8)	2 (5.3)	
BMI (kg/m^2^)	16.4 ± 3.3	16.4 ± 3.5	16.4 ± 2.8	0.977
Family history of DCM, *n* (%)	10 (8.3)	9 (10.8)	1 (2.6)	0.243
Symptoms and signs, *n* (%)				
tachypnea	95 (78.5)	64 (77.1)	31 (81.6)	0.578
fatigue	93 (76.8)	60 (72.3)	33 (86.8)	0.078
hepatomegaly	69 (57.0)	52 (62.7)	17 (44.7)	0.065
edema	44 (36.4)	37 (44.6)	7 (18.4)	
chest distress	42 (34.7)	31 (37.3)	11 (28.9)	0.368
Duration of symptoms (months)	1.0 (0.5–3.0)	1.0 (0.5–2.0)	1.0 (0.5–3.3)	0.464
Duration in first hospitalization (days)	9.0 (6.0–15.0)	8.0 (6.0–16.0)	10.0 (7.0–14.3)	0.194
Multiple hospitalizations, *n* (%)	51 (42.1)	35 (42.2)	16 (42.1)	0.995
Admission to ICU, *n* (%)	53 (43.8)	45 (54.2)	8 (21.1)	0.001
HR at admission (bpm)	113.4 ± 21.9	115.4 ± 21.6	109.2 ± 22.4	0.148
SBP at admission (mmHg)	99.3 ± 14.4	96.6 ± 13.8	105.4 ± 12.9	0.001
DBP at admission (mmHg)	64.8 ± 11.5	63.0 ± 11.2	68.9 ± 10.6	0.007
Cardiac function class, *n* (%)				<0.001
I–II	39 (32.2)	18 (21.7)	21 (55.3)	
III–IV	82 (67.8)	65 (78.3)	17 (44.7)	
Medications at diagnosis, *n* (%)				
Loop diuretic	114 (94.2)	81 (97.6)	33 (86.7)	0.053
Digoxin	114 (94.2)	80 (96.4)	34 (89.5)	0.275
Spironolactone	111 (91.7)	77 (92.8)	34 (89.5)	0.798
ACEIs	103 (85.1)	71 (85.5)	32 (82.2)	0.848
Intravenous inotropes	61 (50.4)	46 (55.4)	15 (39.5)	0.103
β-blocker	37 (30.5)	24 (28.9)	13 (34.2)	0.557
Follow-up time (months)	10.0 (3.0–36.5)	7.0 (2.0–16.0)	64.5 (20.5–108.0)	<0.001

The data are presented as the mean ± standard deviation, median (interquartile range), or number (percentage) based on the type of data. ACEIs, angiotensin-converting enzyme inhibitors; BMI, body mass index; DCM, dilated cardiomyopathy; ICU, intensive care unit; HR, heart rate; SBP, systolic blood pressure; DBP, diastolic blood pressure.

**Table 2:** Laboratory and echocardiographic examinations of patients with DCM.

Parameters	All Patients (*n* = 121)	Death (*n* = 83)	Survivors (*n* = 38)	*p*-Value
Echocardiographic parameters				
LVEDD z score	7.36 ± 2.73	7.67 ± 2.67	6.69 ± 2.80	0.067
LVESD z score	10.42 ± 3.35	10.85 ± 3.12	9.49 ± 3.67	0.038
LVFS z score	−9.45 (−11.55 to −6.36)	−9.45 (−11.9 to −6.70)	−8.79 (−10.85 to −4.72)	0.251
LVEF z score	−6.58 ± 2.17	−6.81 ± 2.04	−6.09 ± 2.38	0.090
LVEDPWT z score	0.60 (−0.59 to 1.67)	0.6 (−0.23 to 1.65)	0.69 (−1.02 to 2.08)	0.521
LVEDST z score	0.11 (−1.03 to 1.14)	0.11 (−1.01 to 1.22)	0.32 (−1.18 to 1.13)	0.692
Laboratory data				
WBC (×10^12^/L)	9.11 (7.70–11.00)	9.10 (7.88–11.33)	9.23 (7.13–10.41)	0.257
hemoglobin (g/L)	125.50 (114.00–137.15)	125.50 (114.00–136.00)	127.75 (113.00–141.93)	0.915
RDW	0.16 (0.15–0.18)	0.16(0.15–0.18)	0.15(0.14–0.17)	0.346
CK-MB (U/L)	18.00 (11.00–36.00)	17.00 (10.00–28.00)	19.00 (13.80–47.30)	0.102
Troponin I (ng/mL)	0.05 (0.02–0.64)	0.07 (0.02–0.64)	0.04 (0.01–0.642)	0.174
Sodium (mmol/L)	136.20 (134.00–139.00)	136.00 (134.00–139.00)	136.25 (135.28–139.18)	0.355
Potassium (mmol/L)	4.25 (4.25–4.50)	4.25 (3.74–4.53)	4.25 (4.25–4.41)	0.270
Calcium (mmol/L)	2.31 (2.18–2.41)	2.28 (2.15–2.39)	2.37 (2.28–2.490	0.002
BUN (μmol/L)	6.03 (4.73–7.20)	6.00 (4.60–7.28)	6.04 (4.81–7.25)	0.987
Serum creatinine (μmol/L)	56.00 (40.50–76.10)	57.00 (42.00–72.10)	56.00 (38.25–83.50)	0.793
Uric acid (μmol/L)	461.32 ± 185.59	469.18 ± 182.55	444.14 ± 193.44	0.503

The data are presented as the mean ± standard deviation, median (interquartile range), or number (percentage) based on the type of data. BUN, blood urea nitrogen; CK-MB, creatine kinase-MB; DCM, dilated cardiomyopathy; LVEDD, left ventricular end-diastolic dimension; LVESD, left ventricular end-systolic dimension; LVEF, left ventricular ejection fraction, LVFS, left ventricular fractional shortening; LVEDPWT, left ventricular end-diastolic posterior wall thickness; LVEDST, left ventricular end-diastolic septal thickness; RDW, red cell distribution width; WBC, white blood cell.

**Table 3:** Predictors of death in patients with DCM according to Cox regression analysis.

	Univariate Cox Regression Analysis			Multivariate Cox Regression Analysis		
Characteristics	HR	95% CI	*p*-Value	HR	95% CI	*p*-Value
Age at diagnosis, per year	0.994	0.954–1.035	0.763			
Female (reference: male)	1.176	0.764–1.809	0.462			
Cardiac function class III–IV (reference: cardiac function class I–II)	2.516	1.488–4.255	0.001	1.801	1.030–3.149	0.039
Heart rate, per bpm	1.008	0.999–1.018	0.094	1.006	0.995–1.017	0.284
SBP, per mmHg	0.968	0.949–0.987	0.001	0.982	0.956–1.008	0.166
DBP, per mmHg	0.977	0.959–0.966	0.018	0.987	0.960–1.016	0.386
LVEDD z score, per SD	1.049	0.974–1.130	0.203			
LVESD z score, per SD	1.049	0.990–1.111	0.108			
LVEF z score, per SD	0.911	0.829–1.002	0.056	0.962	0.867–1.067	0.466
LVFS z score, per SD	0.988	0.940–1.039	0.642			
Calcium levels, per mmol/L	0.222	0.084–0.574	0.002	0.219	0.084–0.576	0.002
Digoxin (reference: without digoxin)	2.072	0.654–6.565	0.216			
Loop diuretic (reference: without loop diuretic)	3.533	0.868–14.836	0.078	1.780	0.412–7.688	0.440
ACEIs (reference: without ACEIs)	0.967	0.524–1.785	0.915			
β-blocker (reference: without β-blocker)	1.084	0.672–1.748	0.740			

ACEIs, angiotensin-converting enzyme inhibitors; CI, confidence interval; DCM, dilated cardiomyopathy; DBP, diastolic blood pressure; HR, hazard ratio; LVEDD, left ventricular end-diastolic dimension; LVESD, left ventricular end-systolic dimension; LVEF, left ventricular ejection fraction; LVFS, left ventricular fractional shortening; SBP, systolic blood pressure.

## Data Availability

The data presented in this study are available on request from the corresponding author. The data are not publicly available due to privacy reasons.
